# Facile electrochemical determination of acetaminophen at micromolar levels utilizing conjugated bimetallic Co–Zn porphyrin polymer electrodes as sensing platforms†

**DOI:** 10.1039/d5ra00178a

**Published:** 2025-06-23

**Authors:** Xue Cai, Meitong Li, Rui Tao, Xinyu Yun, Xinyu Yang, Jiayue Sun, Chuangyu Wei

**Affiliations:** a Heilongjiang Key Laboratory of Photoelectric Functional Materials, College of Chemistry and Chemical Engineering, Mudanjiang Normal University Mudanjiang 157011 P. R. China xuecai@mdjnu.edu.cn chuangyu_wei@mdjnu.edu.cn

## Abstract

The increasing threat of pharmaceutical pollution to public health and the environment is a critical issue. This research endeavors to tackle the challenge by developing an advanced electrochemical sensor for the accurate detection of acetaminophen (APAP). A highly sensitive electrochemical sensor, based on a porphyrin polymer, was designed for this purpose. The study shows that the bimetallic structure of the polymer significantly enhances the sensing efficiency of micropollutants. By analyzing its electrochemical properties, the sensor achieved an impressive detection limit of 0.46 μM for APAP, with a linear detection range from 4 to 1000 μM. The sensor also demonstrated strong anti-interference capabilities, along with high reproducibility and stability. Furthermore, it exhibited excellent performance in analyzing actual samples. Compared with single-metal polymer materials, bimetallic polymer materials exhibit the lowest charge transfer resistance, rapid electron transfer rates, and large electrochemical active areas, thereby enabling superior sensing capabilities.

## Abstract 2

The increasing threat of pharmaceutical pollution to public health and the environment is a critical issue.

## Graphical abstract

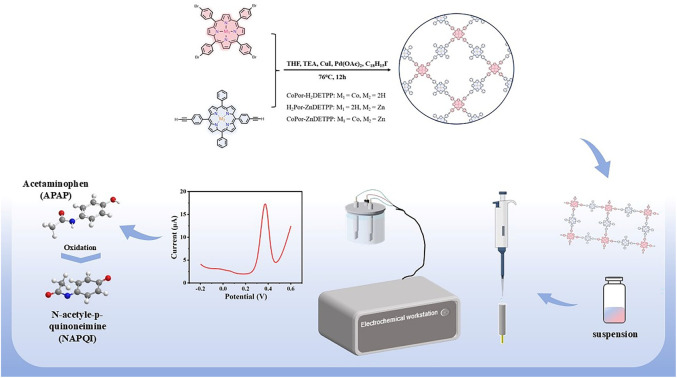

## 1. Introduction

Acetaminophen (APAP) is a commonly used over-the-counter drug, widely employed to relieve pain and reduce fever.^1,2^ However, with its increasing use, the issue of APAP residues in domestic wastewater has gained attention.^3,4^ The main sources of these residues include drug excretion, improper disposal of unused medications, and the discharge of wastewater from hospitals and households. The presence of APAP in water bodies poses potential risks to aquatic organisms and may also impact human health through drinking water.^5,6^ Therefore, developing an efficient and sensitive method for detecting and removing APAP in domestic wastewater is of significant environmental and public health importance. Traditional methods for detecting APAP include liquid chromatography-mass spectrometry (LC-MS),^7^ gas chromatography-mass spectrometry (GC-MS),^8^ high-performance liquid chromatography (HPLC),^9^ fluorescence spectroscopy (FL),^10^ and flow injection analysis (FIA).^11,12^ However, these techniques often require expensive instruments, involve high maintenance costs, necessitate rigorous solvent purification, and have low sensitivity. They can also be cumbersome to operate and are susceptible to numerous interfering factors. In contrast, the electrochemical detection of APAP has garnered increasing attention due to its ease of operation, potential for miniaturization, low cost, and rapid response times.^13,14^ An electrochemical sensor operates by leveraging the electrochemical properties of the target substance, converting chemical information into an electrical signal based on a specific principle. This output signal facilitates the qualitative or quantitative analysis of the target substance.^15,16^ The earliest instance of electrochemical APAP detection dates back to the 1990s.^17^ Since then, diverse electrode materials and modification techniques have been utilized for APAP sensing in electrochemical applications.

In recent years, researchers have developed various electrode materials for APAP detection, including precious metal nanomaterials,^17^ metal oxide materials,^17^ and metal–organic framework-carbon composites.^17^ While these materials have distinct advantages, they also have notable limitations. Precious metal nanomaterials offer a large specific surface area and abundant active sites, enhancing electrochemical reaction rates, but their high cost and complex preparation make them less practical. Metal oxides exhibit high chemical stability across different electrolyte environments, yet their poor electrical conductivity results in significant charge transfer resistance, reducing sensitivity and response speed. Metal–organic framework-carbon composites provide a rich pore structure to facilitate ion diffusion and target substance access to the electrode surface, though their preparation typically involves complex processes.

In recent years, porphyrins have gained significant attention in electrochemical sensing applications, thanks to their unique molecular structure, ease of modification, and distinct optoelectronic properties. For example, Liu *et al.*^18^ synthesized a symmetric metalloporphyrin with copper as the central atom and four carboxyphenyl groups surrounding it (Cu-TCPP), and they polymerized the Cu-TCPP on a glassy carbon electrode. The addition of copper complemented the planar conjugated structure of the porphyrin, providing the new sensor with enhanced sensitivity and stability for detecting bases such as guanine (G). Jemmeli *et al.*^19^ prepared a 5,10,15,20-tetra[(4-methoxyphenyl)]porphyrin cadmium(ii) complex and modified it on carbon paste electrodes to create a novel electrochemical sensor CPE-[Cd(TMPP)], capable of detecting bisphenol A, which has proven effective in real sample analysis. Additionally, Peng *et al.*^20^ developed and studied a biomimetic sensor based on Mn(iii) *meso*-tetra(*N*-methyl-4-pyridyl) porphyrin (MnTMPyP), which has been reliably utilized for the detection of hydrogen peroxide and glucose in human serum samples. Despite these advances, monomolecular porphyrins generally face challenges such as low conductivity, susceptibility to aggregation, and limited chemical stability, which restricts their broader application in electrochemical biosensors.^21–23^ To overcome these limitations, porphyrins are often combined with more conductive materials, such as precious metals (gold and platinum) or carbon-based materials (carbon nanotubes and graphene), to enhance electron transfer kinetics, consequently, improve their electrocatalytic activity toward target analytes.^24,25^ However, the complex structure, self-limiting reactions, unclear active sites, and reduced catalytic selectivity may hinder their suitability for practical applications. Therefore, the development of new sensing materials with high electrochemical activity and well-defined structure for APAP detection is a necessary condition to advance practical applications. Fortunately, porphyrin-based conjugated polymer materials (PCPs) featuring metal–nitrogen coordination centers (M–N_4_) have emerged as promising nanozyme catalysts, owing to their enzyme-like catalytic structure and exceptional catalytic activity. Based on the above, the preparation of porphyrin-based conjugated polymers as electrochemical sensors has attracted much attention, and there are already relevant literature reports.^26–28^ However, the electrochemical sensing performance of APAP biomolecules by bimetallic porphyrin conjugated polymers has not been reported.

**Scheme 1:**
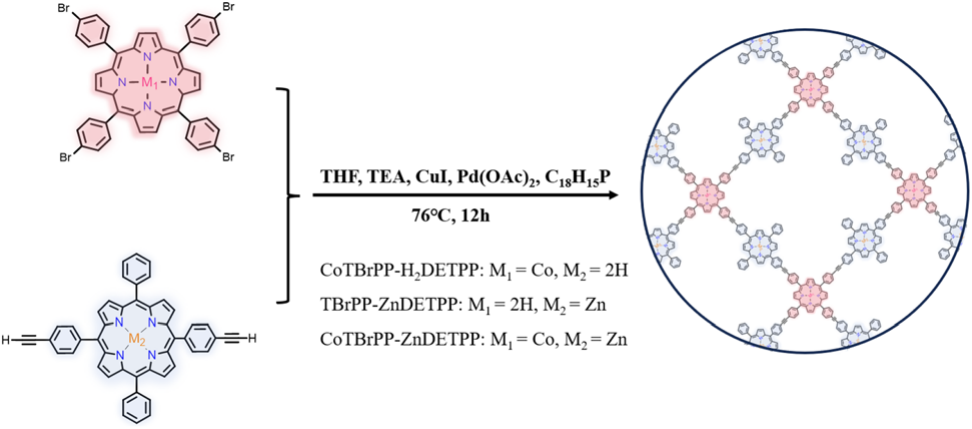
Schematic illustration of the polymers.

In this thesis, a porphyrin conjugated polymer (CoTBrPP-ZnDETPP) was successfully designed and synthesized by the Sonogashira coupling reaction and based on this, the electrochemical sensing platform (CoTBrPP-ZnDETPP/GCE) was established for detection of APAP, Scheme 1. The morphology, structure, and components of the obtained CoTBrPP-ZnDETPP were confirmed by relative physical characterization. The electrochemical sensing capabilities of CoTBrPP-ZnDETPP/GCE for APAP detection has been studied employing cyclic voltammetry (CV) and differential pulse voltammetry (DPV) techniques. Through the optimization of experimental conditions, the prepared CoTBrPP-ZnDETPP/GCE exhibits high selectivity, good sensitivity, excellent anti-interference ability, low detection limit, and stability for detecting APAP. Additionally, it has been successfully applied to the detection of real samples, yielding acceptable recoveries. The relationship between the structure and sensing properties of CoTBrPP-ZnDETPP will be analyzed and discussed. For comparison, CoTBrPP-H_2_DETPP and TBrPP-ZnDETPP were also prepared and compared. This study will offer a theoretical foundation and technical support for the rapid detection of APAP, contributing significantly to real sample analysis and monitoring.

## 2. Experimental

The compounds 5,10,15,20-tetrakis(4-bromophenyl)-porphyrin (TBrPP), cobalt(ii) 5,10,15,20-tetrakis(4′-bromophenyl)-porphyrin (CoTBrPP), 5,15-di(4-ethynylphenyl)-10,20-diphenylporphyrin (H_2_DETPP) and zinc(ii) 5,15-di(4-ethynylphenyl)-10,20-diphenylporphyrin (ZnDETPP) were synthesized following the method described in Fig. S1 and S2† (ref. 29) and characterized using spectroscopic techniques, including ^1^H NMR spectroscopy and UV-vis absorption spectra (Fig. S3–S7†). CoTBrPP-ZnDETPP was synthesized *via* a Sonogashira coupling reaction, as illustrated in Scheme 1. To a flask containing CoTBrPP (12.8 mg, 0.013 mmol) and ZnDETPP (20.4 mg, 0.028 mmol), a NEt_3_/THF solution (40 mL, 1 : 3 v/v) was added, which was purged with nitrogen for 30 minutes. Subsequently, PPh_3_ (29 mg, 0.110 mmol), Pd(OAc)_2_ (6.3 mg, 0.028 mmol) and CuI (10.5 mg, 0.055 mmol) were introduced into the mixture. The reaction was performed at 76 °C with stirring for 12 hours, followed by filtration and sequential washing with THF, H_2_O, CH_3_OH, CHCl_3_ and C_3_H_6_O. The residue was then vacuum-dried at room temperature, yielding CoTBrPP-ZnDETPP. The synthesis of CoTBrPP-H_2_DETPP and TBrPP-ZnDETPP followed the same procedure, preparation of modified electrode and electrochemical measurements in ESI.†

## 3. Results and discussion

### 3.1 Structural characterization of monomers and polymers

**Fig. 1:**
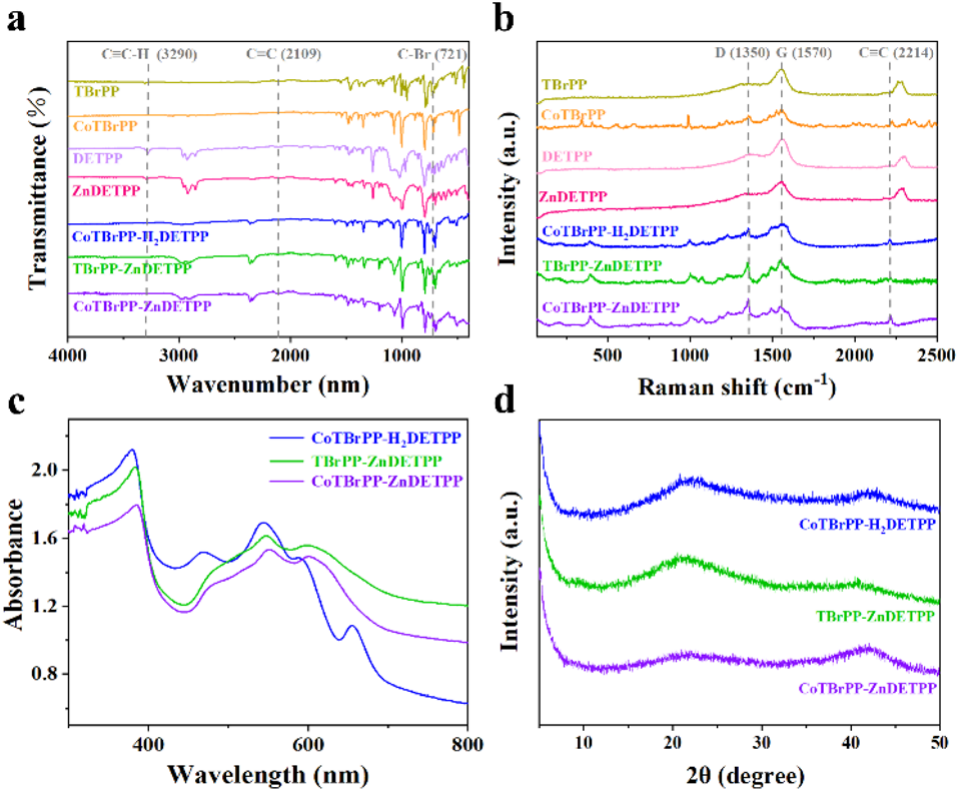
FT-IR spectra (a), Raman spectra (b), UV-vis absorption spectra (c), and XRD pattern (d) of polymers and the corresponding porphyrin monomers.

In the Fourier transform infrared (FT-IR) spectra (Fig. 1a), both the monomers (TBrPP, CoTBrPP, H_2_DETPP and ZnDETPP) and the polymers (CoTBrPP-H_2_DETPP, TBrPP-ZnDETPP and CoTBrPP-ZnDETPP) showed comparable infrared features, in agreement with the literature report.^30,31^ The CoTBrPP and TBrPP monomers at ∼721 cm^−1^ show C–Br vibration, however, it disappears in three polymers. In addition, it can be seen that in three polymers, the C≡C–H vibrational peak at ∼3290 cm^−1^ vanished and the C≡C vibrational peak at ∼2109 cm^−1^ remained, confirms the successful incorporation of the alkynyl group into the structure of CoTBrPP-H_2_DETPP, TBrPP-ZnDETPP and CoTBrPP-ZnDETPP.^29,30^ Additionally, as shown in the Raman spectrum (Fig. 1b), the C≡C stretching peak around ∼2214 cm^−1^ was observed in all three polymers, but was absent in the H_2_DETPP and ZnDETPP monomers.^30^ Those evidence presented above suggests that the three porphyrin polymers were successfully synthesized. Additionally, a defect-related D-band at ∼1350 cm^−1^ and a broad G-band at ∼1570 cm^−1^ are observed, representing the disordered graphitic carbon and the degree of graphitization, respectively. The intensity ratio of *I*_D_/*I*_G_ increases from 0.56 (CoTBrPP-H_2_DETPP) and 0.85 (TBrPP-ZnDETPP) increased to 0.88 (CoTBrPP-ZnDETPP), which indicates the presence of higher defects in the bimetallic CoTBrPP-ZnDETPP. The increase in defects can enhance the material's adsorption capacity for target molecules, providing more interaction interfaces for electrochemical reactions, thereby contributing to improved sensing performance.

The UV-vis diffuse reflectance spectra (DRS) of TBrPP, H_2_DETPP, and their metal complexes CoTBrPP and ZnDETPP were showed in Fig. S8.† Moreover, the DRS of the CoTBrPP-H_2_DETPP, TBrPP-ZnDETPP and CoTBrPP-ZnDETPP (Fig. 1c) exhibited a combination of the absorption features characteristic of their corresponding monomers, meanwhile notable red-shifts for the Soret bands were observed from 369 and 362 nm in CoTBrPP and ZnDETPP monomers to 385 nm in CoTBrPP-ZnDETPP, the Soret bands of CoTBrPP-H_2_DETPP and TBrPP-ZnDETPP were red-shifted to 379 and 383 nm, respectively, compared to their corresponding monomers. This redshift can be attributed to the edge-to-edge stacking of porphyrin units within the polymer structures^31–33^ X-ray diffraction (XRD) results indicated that the crystallinity was significantly decreased upon polymerization and transformed into an amorphous structure (Fig. 1d).^29,34^ The above findings demonstrate the successful synthesis of the three polymers.

**Fig. 2:**
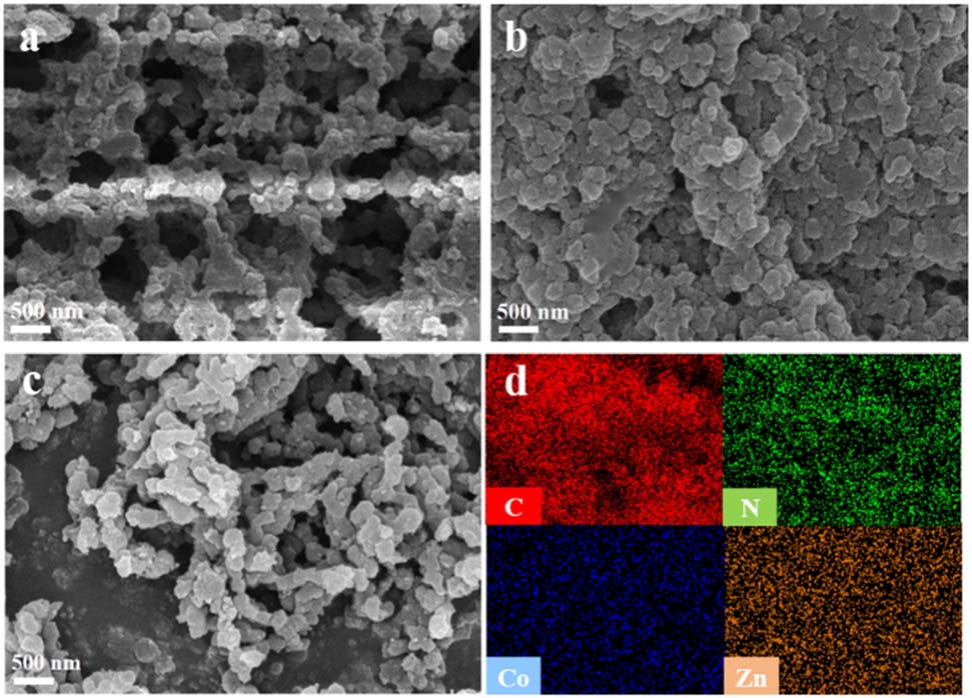
SEM image of CoTBrPP-H_2_DETPP (a), TBrPP-ZnDETPP (b), CoTBrPP-ZnDETPP (c), and corresponding EDS mapping images of C, N, Co and Zn (d).

Scanning electron microscopy (SEM) was used to analyze the microscopic morphology of CoTBrPP-H_2_DETPP, TBrPP-ZnDETPP, and CoTBrPP-ZnDETPP, as illustrated in Fig. 2a–c. All three polymers exhibited a highly porous and granular structure with consistent uniformity,^35^ promoting the exposure of additional active sites and enhancing redox reactions on the electrode surface. Additionally, energy dispersive spectrometer (EDS) mapping analysis confirmed the uniform distribution of C, N, Co, and Zn elements on the surface of CoTBrPP-ZnDETPP, as shown in Fig. 2d, further validating the successful synthesis of CoTBrPP-ZnDETPP.

**Fig. 3:**
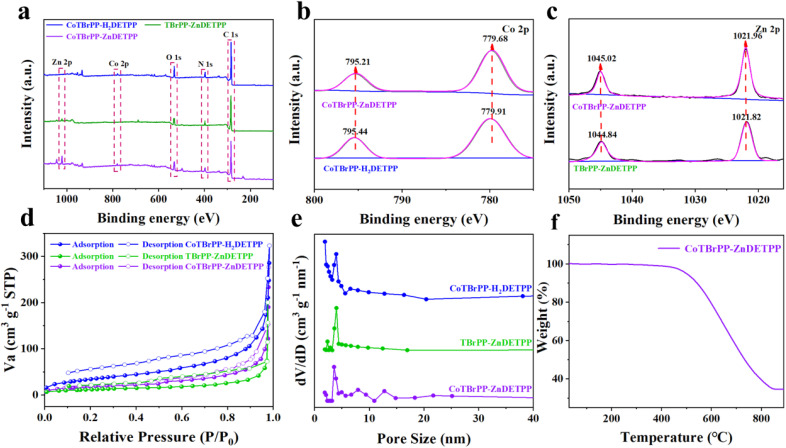
Survey XPS spectra (a), comparison of Co 2p XPS spectra (b), comparison of Zn 2p XPS spectra (c), nitrogen adsorption–desorption isotherms (d), pore size distributions (e), and TGA (f) of CoTBrPP-H_2_DETPP, TBrPP-ZnDETPP and CoTBrPP-ZnDETPP.

Fig. 3a shows the full X-ray photoelectron spectroscopy (XPS) spectrum of CoTBrPP-H_2_DETPP, TBrPP-ZnDETPP and CoTBrPP-ZnDETPP, which further reveals the presence of Zn, Co, O, N and C elements. Among them, the element O, which comes from water and oxygen in the air, was adsorbed the most due to the largest specific surface area of CoTBrPP-H_2_DETPP, and conversely TBrPP-ZnDETPP the least, which is also in agreement with the specific surface area results.^36,37^ And the Co 2p high-resolution spectra of CoTBrPP-H_2_DETPP (Fig. 3b) could be fitted to two asymmetric peaks with binding energies of 779.91 (Co 2p_3/2_) and 795.44 (Co 2p_1/2_) eV, which are in general agreement with the theoretical binding energy of Co(ii), thus indicating that the valence state of Co is +2. The high-resolution XPS spectra of Zn 2p of TBrPP-ZnDETPP (Fig. 3c) showed two binding energies of 1021.82 (Zn 2p_3/2_) and 1044.84 (Zn 2p_1/2_) eV peaks, which are in general agreement with the theoretical binding energy of Zn(ii), thus indicating that the valence state of Zn is +2. In the CoTBrPP-ZnDETPP polymer, the binding energies of Co are 779.68 (Co 2p_3/2_) and 795.21 (Co 2p_1/2_) eV, respectively, which are more negative than that of the monometallic polymer CoTBrPP-H_2_DETPP by negatively shifted by about 0.23 eV; the binding energies of Zn were 1021.96 (Zn 2p_3/2_) and 1045.02 (Zn 2p_1/2_) eV, respectively, which were positively shifted by about 0.15 eV than that of the monometallic polymer TBrPP-ZnDETPP. These XPS results indicated that electron transfer exists in CoTBrPP-ZnDETPP,^29^ which makes this material particularly advantageous for sensing.

To investigate the specific surface area and porosity of CoTBrPP-H_2_DETPP, TBrPP-ZnDETPP and CoTBrPP-ZnDETPP, N_2_ sorption isotherm experiments at 77 K were performed (Fig. 3d and e). The three polymers displayed comparable type IV adsorption–desorption isotherms, indicating the presence of microporous or mesoporous structures. The adsorption and desorption curves exhibited a distinct hysteresis loop characteristic of the H4 type.^38^Table 1 summarises the specific surface areas of CoTBrPP-H_2_DETPP, TBrPP-ZnDETPP and CoTBrPP-ZnDETPP as 125.37 m^2^ g^−1^, 42.69 m^2^ g^−1^ and 63.22 m^2^ g^−1^, with average pore sizes of 15.9549 nm, 29.0330 nm and 27.0728 nm, respectively. Although the specific surface area of CoTBrPP-ZnDETPP is slightly reduced due to the coordination of the second metal, Zn, the bimetal's excellent biocompatibility and good electrical conductivity may offer more active sites and facilitate a faster charge transfer rate, leading to superior electroanalytical performance. To better understand the thermal stability of bimetallic polymers, thermogravimetric analysis (TGA) wasconducted on CoTBrPP-ZnDETPP and its monomers, CoTBrPP and ZnDETPP, under a nitrogen (N_2_) atmosphere, as shown in Fig. 3f and S9.† The results indicate that the polymers exhibit significantly improved thermal stability compared to their respective monomers. Below 445 °C, apart from a minor loss attributed to the evaporation of residual solvent and water, the bimetallic polymer CoTBrPP-ZnDETPP remained thermally stable with no significant decomposition. However, above 445 °C, decomposition of the polymer became evident, with a pronounced weight loss observed. At approximately 845 °C, the total weight loss reached nearly 65%, primarily due to the decomposition of the metal porphyrin framework. These findings demonstrate that the synthesized bimetallic porphyrin conjugated polymers possess excellent thermal stability.

**Table 1:** Specific surface area of different polymers

Polymers	Specific surface area (m^2^ g^−1^)	Pore volume (cm^3^ g^−1^)	Average pore sizes (nm)
CoTBrPP-H_2_DETPP	125.37	0.5001	15.9549
TBrPP-ZnDETPP	42.69	0.3099	29.0330
CoTBrPP-ZnDETPP	63.22	0.4279	27.0728

### 3.2 Electrochemical characterization

**Fig. 4:**
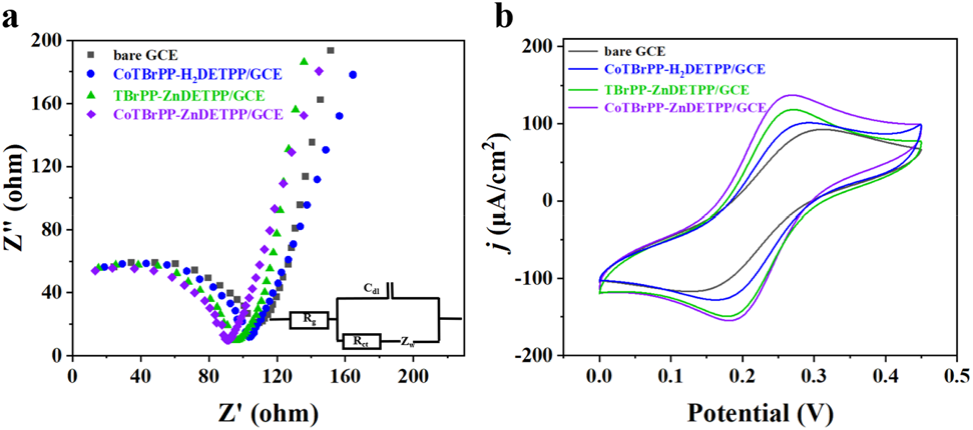
EIS, inset is the Randles equivalent circuit (a), and CVs (b) of bare GCE, CoTBrPP-H_2_DETPP/GCE, TBrPP-ZnDETPP/GCE and CoTBrPP-ZnDETPP/GCE in 0.1 M KCl containing 1 mM [Fe(CN)_6_]^3−/4−^.

EIS is an essential method for evaluating the interfacial and electron transfer characteristics of surface-modified electrodes. Fig. 4a presents the Nyquist plots of various modified electrodes in 0.1 M KCl solutions containing 1 mM [Fe(CN)_6_]^3−/4−^. The impedance (*R*_ct_) values of individual electrodes were obtained by modeling the electrical network through Randles equivalent circuit (Fig. 4a, inset) using Zview software. It is well established that the charge transfer resistance (*R*_ct_) is represented by the diameter of the semicircle, which indicates the electron transfer dynamics at the electrode interface. A narrower radius indicates less resistance to charge transfer and *vice versa*.^39,40^ As revealed in Table 2, the *R*_ct_ values of GCE, CoTBrPP-H_2_DETPP/GCE, TBrPP-ZnDETPP/GCE and CoTBrPP-ZnDETPP/GCE were 109.10, 104.17 95.63 and 90.76 Ω, respectively. In comparison to GCE, CoTBrPP-H_2_DETPP/GCE and TBrPP-ZnDETPP/GCE, the electrochemical impedance curve of CoTBrPP-ZnDETPP/GCE displays the minimal semicircle radius in the high-frequency domain, signifying the highest charge transfer rate and the fastest electrocatalytic reaction kinetics. This observation highlights that the incorporation of bimetals enhances the electrical conductivity of CoTBrPP-ZnDETPP, thereby promoting the associated interfacial electrochemical reactions and enabling the potential for high sensitivity.

**Table 2:** *I*_p_, EASAs, and *R*_ct_ of bare GCE, CoTBrPP-H_2_DETPP/GCE, TBrPP-ZnDETPP/GCE and CoTBrPP-ZnDETPP/GCE

Materials	*I*_p_ (μA)	EASAs (cm^2^)	*R*_ct_ (Ω)
bare GCE	6.54	0.0059	109.10
CoTBrPP-H_2_DETPP	7.13	0.0065	104.17
TBrPP-ZnDETPP	8.36	0.0076	95.63
CoTBrPP-ZnDETPP	9.75	0.0089	90.76

The electrochemical characteristics of various modified electrodes were investigated using CV with [Fe(CN)_6_]^3−/4−^ as a redox probe (Fig. 4b). Of all the electrodes tested, CoTBrPP-ZnDETPP/GCE showed the strongest redox signal, displaying the highest peak oxidation current density (138.99 μA cm^−2^) and the smallest redox potential difference (Δ*E*_p_) of 84 mV. The intensity of the peak current indicates the extent of the electrochemically active surface areas (EASAs) and the efficiency of electron transfer. According to the RandlessSevcik equation, *I*_p_ = 2.69 × 10^5^*AD*^1/2^*n*^2/3^*V*^1/2^*C*, the EASAs of bare GCE, CoTBrPP-H_2_DETPP/GCE, TBrPP-ZnDETPP/GCE and CoTBrPP-ZnDETPP/GCE were approximately 0.0059 cm^2^, 0.0065 cm^2^, 0.0076 cm^2^ and 0.0089 cm^2^. The results indicate that CoTBrPP-ZnDETPP/GCE possesses the largest electrochemically active surface area, thus demonstrating the highest electrochemical activity, which aligns well with the EIS findings.

### 3.3 Sensing properties towards APAP

**Fig. 5:**
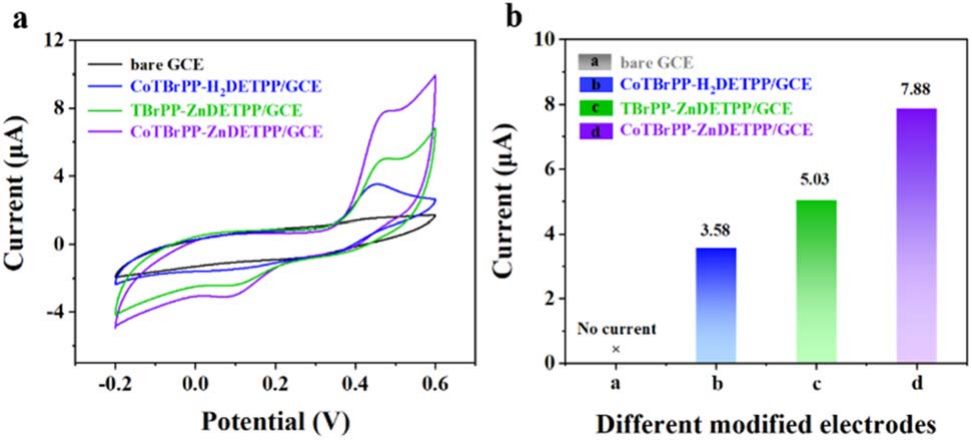
CVs detection curves of bare GCE, CoTBrPP-H_2_DETPP/GCE, TBrPP-ZnDETPP/GCE and CoTBrPP-ZnDETPP/GCE electrode in 0.1 M PBS buffer solution containing 0.4 mM APAP (a), and correlated bar graph diagram of peak current *versus* different modified electrodes (b).

The electrochemical behavior of CoTBrPP-H_2_DETPP, TBrPP-ZnDETPP and CoTBrPP-ZnDETPP was assessed to determine their potential as electrochemical sensors. To ensure that all steps in the construction of the electrochemical sensor were successfully completed, CV of the variously modified electrodes in a 0.1 M PBS solution containing 0.4 mM APAP was performed to evaluate the stepwise construction process. In Fig. 5a, the CV the CoTBrPP-ZnDETPP modified GCE exhibits a superior current response in comparison to other GCEs upon the addition of APAP. Compared to the bare GCE and the electrodes modified with CoTBrPP-H_2_DETPP and TBrPP-ZnDETPP, which exhibited low peak current responses (*I*_pa_) of 0 μA, 3.58 μA and 5.03 μA, respectively, the CoTBrPP-ZnDETPP modified electrode demonstrated a distinct catalytic oxidation peak with a notably higher current response (*I*_pa_) of 7.88 μA when APAP was present. The CoTBrPP-ZnDETPP-modified electrode demonstrates excellent performance in the electro-oxidation of APAP. The corresponding bar graph of peak current *versus* electrode material is shown in Fig. 5b. Under the same conditions, bare GCE and CoTBrPP-ZnDETPP electrodes were tested for CV in PBS solution, as shown in Fig. S10.† No significant redox peaks were observed, indicating that PBS does not interfere with the APAP test. The CoTBrPP-ZnDETPP-modified electrode is employed for further electrochemical investigations into APAP detection.

**Fig. 6:**
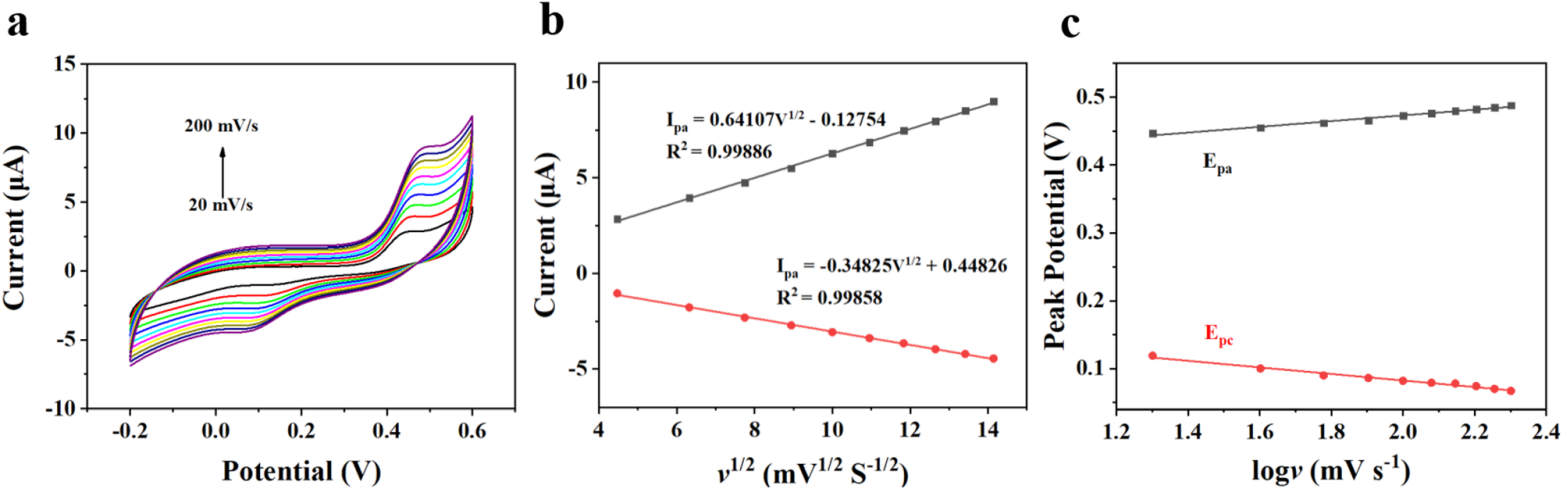
(a) CVs of CoTBrPP-ZnDETPP/GCE in 0.1 M PBS with 0.4 mM APAP at different scan rates, (b) the relationship between redox peak currents of APAP and *v*^1/2^, and (c) the relationship between redox peak potentials and log *v*.

Additionally, the mechanism of the APAP reaction on CoTBrPP-ZnDETPP/GCE was investigated by varying the scan rate. As shown in Fig. 6a, both the oxidation and reduction peak currents increased as the scan rate was increased from 20 to 200 mV s^−1^. An excellent linear correlation between peak current (*I*_p_) and the square root of scan rates (*v*^1/2^) can be obtained (Fig. 6b), and the linear equations were *I*_pa_ = 0.64107*v*^1/2^ (mV^1/2^ s^−1/2^) + 0.12754, *R*^2^ = 0.99886 and *I*_pc_ = −0.34825*v*^1/2^ (mV^1/2^ s^−1/2^) + 0.44826, *R*^2^ = 0.99858. These results indicate that the electro-oxidation reaction of APAP on the electrode surface is a diffusion-controlled process.^41–43^

**Scheme 2:**
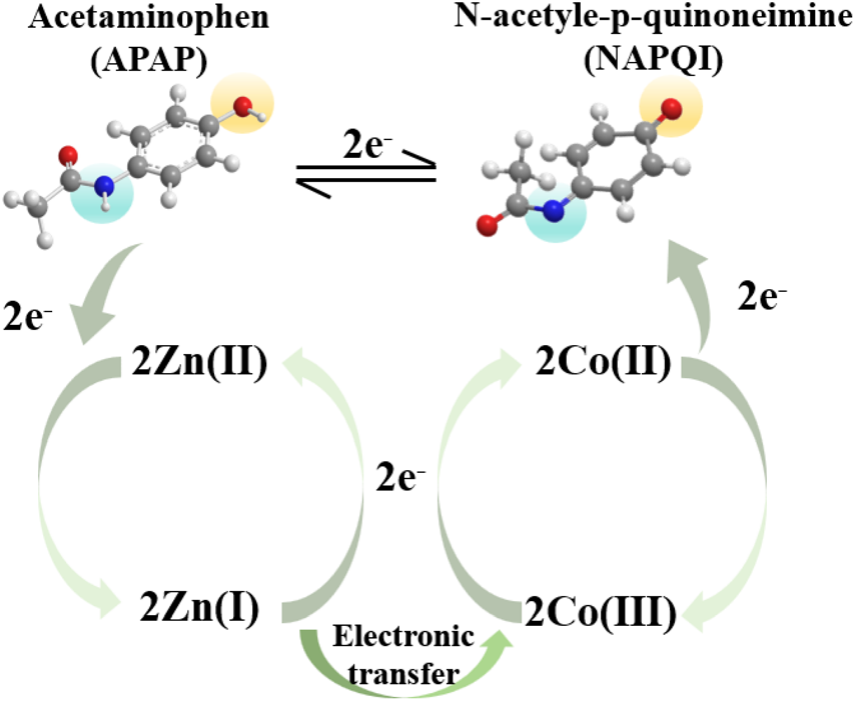
Reaction mechanism for APAP at CoTBrPP-ZnDETPP/GCE.

Fig. 6c illustrates the correlation between the logarithm of the scan rate (log *v*) and the peak potentials (*E*_pa_ and *E*_pc_). They can be provided as follows: *E*_pa_ = 0.04215 log *v* (mV s^−1^) + 0.38892, *R*^2^ = 0.98 and *E*_pc_ = −0.0484 log *v* (mV s^−1^) + 0.17922, *R*^2^ = 0.98. The Laviron theory^44^ was used to analyze the redox mechanism of APAP molecules occurring on CoTBrPP-ZnDETPP/GCE. According to theory, the slopes of the anodic and cathodic peak curves are given by 2.3*RT*/(1 − *α*)*nF* and −2.3*RT*/*αnF*,^45,46^ respectively, where *n* denotes the number of electrons involved in the process, *R* stands for the gas constant, and *F* represents the Faraday constant. The calculated charge transfer coefficient, *α*, was estimated to be around 0.5, and *n* was found to be around 2 (with *R* = 8.314, *T* = 298, and *F* = 96 480). In the APAP oxidation process, an equivalent amount of protons and electrons participate, indicating that the CoTBrPP-ZnDETPP/GCE follows a two-electron and two-proton process. This result is also consistent with that reported in the literature.^47^ Meanwhile, the electrochemical parameters (*K*_s_) can be calculated by formulas (1)–(4). It is known that *n* is about 2, the variables *R*, *F*, and *T* are constants under standard conditions, and the slopes of the *E*_pa_ and *E*_pc_ equations are 0.06018 and −0.08369, respectively. The value of *K*_s_ for CoTBrPP-ZnDETPP/GCE is 2.53 cm s^−1^, which is larger than the 1.45 cm s^−1^ for CoTBrPP-H_2_DETPP/GCE and the 2.18 cm s^−1^ for TBrPP-ZnDETPP/GCE, suggesting that faster electron transfer between APAP and CoTBrPP-ZnDETPP/GCE was achieved. Based on these findings, Scheme 2 shows the following mechanism.1
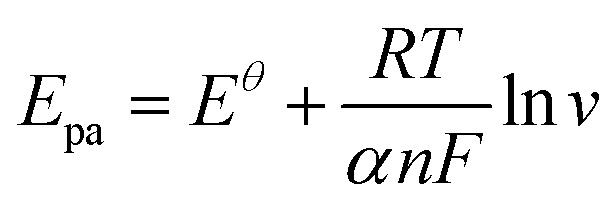
2
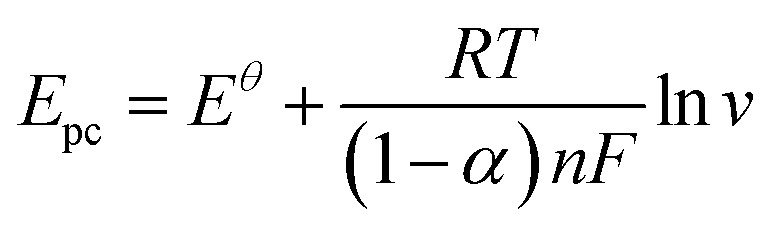
3
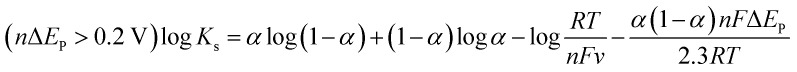
4
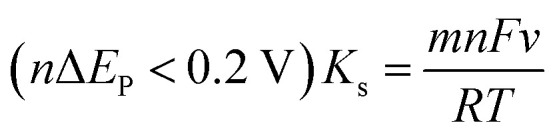


### 3.4 Analytical performance of CoTBrPP-ZnDETPP/GCE sensor for APAP detection

The amount of material applied in our study was optimized through DPV analysis. As shown in Fig. S11,† varying volumes (4, 6, 8, 10 and 12 μL) of CoTBrPP-ZnDETPP were applied to the GCE surface, and their corresponding DPV results were analyzed. The oxidation peak current response of APAP increased clearly with volumes from 4 to 10 μL but decreased when the volume increased from 10 to 12 μL. At a 12 μL loading of CoTBrPP-ZnDETPP/GCE, the oxidation response diminished due to the excessive mass load on the GCE surface, which hindered mass and electron transfer at the electrode-material interface. These findings indicate that the optimal APAP oxidation peak electrochemical studies.

**Fig. 7:**
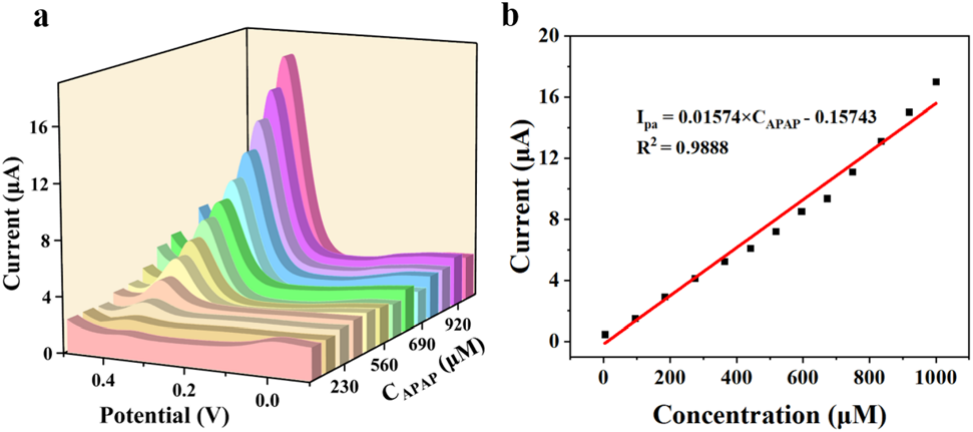
The DPV signals at CoTBrPP-ZnDETPP/GCE of APAP with different concentrations (4–1000 μM) in PBS (a), and the peak current intensity *vs.* The concentrations of APAP (b).

The sensing performance of the CoTBrPP-ZnDETPP/GCE was evaluated by measuring the current responses to different APAP concentrations. The DPV technique, which offers superior sensitivity and resolution compared to CV measurements,^48^ was used to assess the electrocatalytic activity of CoTBrPP-ZnDETPP/GCE towards APAP under optimal detection conditions. The response current showed a gradual increase with the APAP concentration ranging from 4 μM to 1000 μM (Fig. 7a), demonstrating a linear relationship described by the equation *I*_pa_ = 0.01574*C*_APAP_ (μM) − 0.15743, with *R*^2^ = 0.9888 (Fig. 7b). Additionally, the limit of detection (LOD) was determined to be 0.46 μM (S/N = 3), and the sensitivity of CoTBrPP-ZnDETPP/GCE was calculated to be 15.74 mA μM^−1^ cm^−2^.^49^ When compared to other reported works in Table 3, these results highlight the outstanding electroanalytical performance of the CoTBrPP-ZnDETPP/GCE sensor for APAP detection. In addition, we tested CoTBrPP-ZnDETPP/GCE (4–400 μM) and TBrPP-ZnDETPP/GCE (100–400 μM) at different concentrations of DPV (Fig. S12†) and calculated the LOD to be 5.36 μM. The results of the tests showed that the bimetallic polymers possessed a much lower detection concentration and detection limit.

**Table 3:** Comparison of reported performance of modified electrodes for detection of APAP response^a^

Modified electrode	Linear range (μM)	Detection limit (μM)	Reference
CoPc/MCNT	1–1000	1	50
GCE/AuNPs/ZIF-L	5–500	1.02	51
Au/ZIF8	3.5–56	1.02	52
Pt-Co/NPs/3,4,DHPID/CPE	1–850	0.6	53
CoTBrPP-ZnDETPP	4–1000	0.46	This work

a Abbreviations: CoPc-Co phthalocyanines; MCNT-carbon nanotube; ZIF-L-leaf-like zeolitic imidazolate framework; 3,4,DHPID-2-(3,4-dihydroxyphenethyl)isoindoline-1,3-dione; CPE-carbon paste electrode.

### 3.5 Reproducibility, selectivity, interference and stability detection

**Fig. 8:**
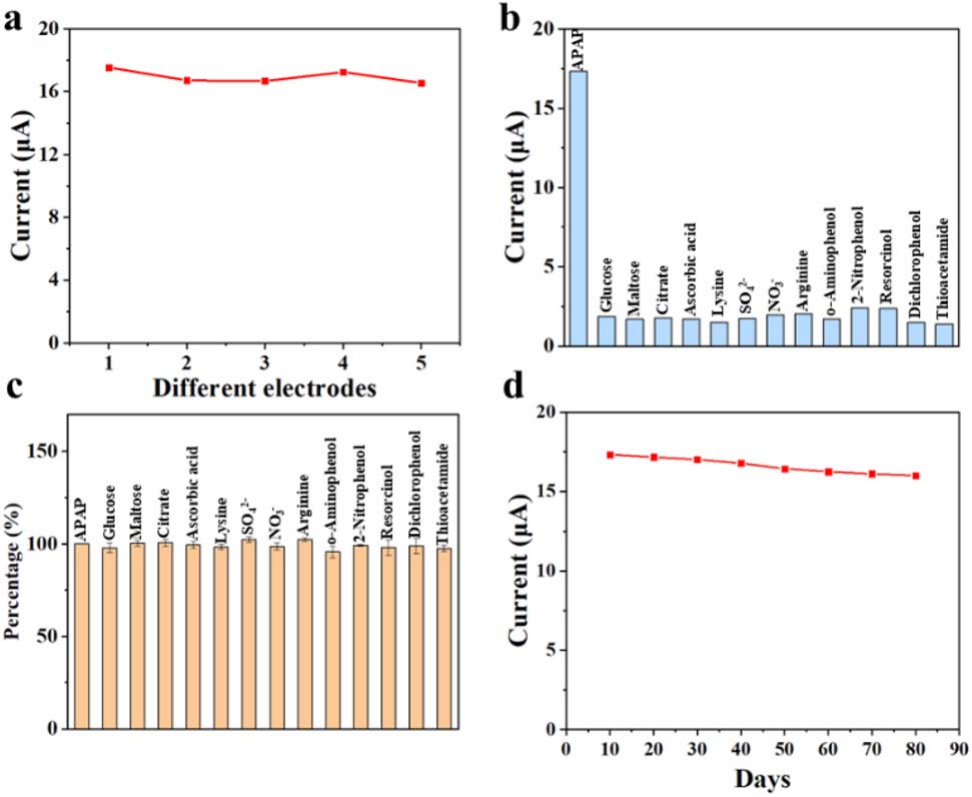
The reproducibility (a), selectivity (b), interference (c), and stability (d) of CoTBrPP-ZnDETPP/GCE sensor.

Reproducibility, selectivity, interference and stability are important indicators to evaluate the sensing performance of electrochemical sensors. Fig. 8a demonstrates the reproducibility test for CoTBrPP-ZnDETPP/GCE with a relative standard deviation (RSD) of 2.66%. To demonstrate and validate the ability of the CoTBrPP-ZnDETPP/GCE for selective APAP detection, the effects of several potential interfering substances were studied under identical conditions. Common biomolecules that coexist with APAP include glucose, maltose, citrate, lysine, SO_4_^2−^, NO_3_^−^, ascorbic acid and arginine. Additionally, some structurally similar substances to APAP are *o*-aminophenol, 2-nitrophenol, resorcinol, dichlorophenol and thioacetamide. The CoTBrPP-ZnDETPP/GCE sensor exhibits a response current to APAP that is more than five times higher than that to other substances, as shown in Fig. 8b. Furthermore, DPV curves of a mixed solution containing APAP and the aforementioned 13 substances were tested using the CoTBrPP-ZnDETPP/GCE sensor. The concentration of the interfering agents was 10 times higher than that of APAP. Despite the high concentration of interfering substances, the detection system still showed a distinct current response to APAP, as illustrated in Fig. 8c. Further, we examined the CV curves of GCE for various substances (Fig. S13†), and the results demonstrated that GCE did not exhibit any response to any of the substances. This result confirms that our constructed electrochemical sensor has high selectivity and anti-interference ability. Moreover, the current response to APAP was maintained at 91.85% of its initial value by detecting the CoTBrPP-ZnDETPP/GCE after 80 days of storage in sample bottles at ambient temperature (Fig. 8d). The good long-term stability of our constructed electrochemical sensor was confirmed. Additionally, the DPV data involved in the Fig. S14.†

### 3.6 Determination of APAP in real samples

To validate the application of the constructed electrochemical sensor in practical detection, the results of APAP detection in urine and wastewater near the hospital were studied. All experiments were performed in accordance with the Guidelines of Mudanjiang Normal University. Informed consents were obtained from human participants of this study (Urine samples). The results are shown in Fig. S15† and Table 4. The spiked recovery rates of APAP ranged from 98.4% to 100.39%, with RSD < 3.1%. Each measurement was performed in triplicate. The results indicate that the constructed electrochemical sensor exhibits reliable and sensitive analytical performance in the analysis of real samples.

**Table 4:** Determination of APAP at electrode in real samples (*n* = 3)

Real samples	Added (μM)	Found (μM)	Recovery (%)	RSD (%)
Urine	50	49.02	98.4	2.3
	100	99.76	99.76	1.9
	200	200.35	100.18	2.7
Wastewater	50	49.45	98.9	2.6
	100	99.52	99.52	2.4
	200	200.77	100.39	3.1

## 4. Conclusion

In conclusion, the bimetallic porphyrin polymer (CoTBrPP-ZnDETPP) was synthesized through the Sonogashira coupling reaction and exhibited clearly enhanced activity owing to its distinctive structure and the synergistic effect of highly exposed bimetallic active sites. The prepared sensor's analytical performance for the sensitive and selective detection of APAP was successfully validated in both laboratory solutions and real sample analyses. When optimized conditions were applied, the detection limit was determined to be 0.46 μM, the quantification limit was 1.53 μM, and the sensitivity was measured at 15.74 mA μM^−1^ cm^−2^. The main benefit of the developed sensor lies in its straightforward and cost-effective electrode modifier preparation, high sensitivity, selectivity and reproducibility. These findings suggest that the CoTBrPP-ZnDETPP sensor has the potential to be developed into a simple, rapid, practical, and effective electrochemical platform for environmental analysis applications.

## Author contributions

Xue Cai: writing – original draft, funding acquisition. Meitong Li: investigation, data curation. Rui Tao: investigation, data curation. Xinyu Yun: investigation. Xinyu Yang: investigation. Jiayue Sun: data curation. Chuangyu Wei: writing – review & editing, supervision and funding acquisition.

## Conflicts of interest

There are no conflicts to declare.

## Supplementary Material

RA-015-D5RA00178A-s001

## Data Availability

The data are available from the corresponding author on reasonable request.
