# Supplementary material for: Facile electrochemical determination of acetaminophen at micromolar levels utilizing conjugated bimetallic Co–Zn porphyrin polymer electrodes as sensing platforms
Source: RSC Adv. 2025 Jun 23;15(26):21190–8. doi: 10.1039/d5ra00178a (PMC12184089; doi:10.1039/d5ra00178a)
Supplement: RA-015-D5RA00178A-s001 [file RA-015-D5RA00178A-s001.pdf]

Supplementary Information (SI) for RSC Advances.
This journal is © The Royal Society of Chemistry 2025

Supporting Information

Facile electrochemical determination of acetaminophen at micromolar levels utilizing conjugated bimetallic Co-Zn porphyrin polymer electrodes as sensing platform

Xue Cai*, Meitong Li, Rui Tao, Xinyu Yun, Xinyu Yang, Jiayue Sun, Chuangyu Wei*

Heilongjiang Key Laboratory of Photoelectric Functional Materials, College of Chemistry and Chemical Engineering, Mudanjiang Normal University, Mudanjiang, 157011, P. R. China

* Corresponding author

E-mail: xuecai@mdjnu.edu.cn and chuangyu_wei@mdjnu.edu.cn

Experimental Section

Chemical and reagents

p-Nitrobenzaldehyde ($C_7H_5NO_3$, ≥ 97%), propionic acid ($C_3H_6O_2$, ≥ 99.5%), pyrrole (C_4H_5N, ≥ 99%), methanol (CH_4O, ≥ 99.5%), dichloromethane (CH_2Cl_2, ≥ 99.9%), N,N-dimethylformamide (DMF, ≥ 99.5%), cobalt(II) acetate tetrahydrate ($C_4H_6CoO_4·4H_2O$, ≥ 99.5%), benzaldehyde (C_6H_5CHO, ≥ 99%), hydrochloric acid (HCl, 37%), petroleum ether (C_nH_{2n+2}, AR), 4-[(trimethylsilyl)ethynyl]benzaldehyde ($C_{12}H_{14}OSi$, ≥ 97%), trifluoroacetic acid (CF_3COOH, ≥ 99%), 2,3-dichloro-5,6-dicyano-p-benzoquinone ($C_8Cl_2N_2O_2$, ≥ 98%), triethylamine ($C_6H_{15}N$, ≥ 99.5%), anhydrous potassium carbonate (K_2CO_3, ≥ 99%), zine acetate dihydrate ($C_4H_6O_4Zn·2H_2O$, ≥ 99%), tetrahydrofuran (C_4H_8O, ≥ 99.5%), triphenylphosphine ($C_{18}H_{15}P$, ≥ 98%), palladium acetate ($C_4H_6O_4Pd$, ≥ 98%), copper iodide (CuI, ≥ 99%), trichloromethane ($CHCl_3$, ≥ 99%), acetone (C_3H_6O, ≥ 99.5%), sodium sulfate anhydrous (Na_2SO_4, ≥ 99%), ethanol(C_2H_6O, ≥ 98%), potassium dihydrogen phosphate (KH_2PO_4, ≥ 99.5%), potassium chloride (KCl, ≥ 99.5%), disodium hydrogen phosphate dodecahydrate (Na_2HPO_4, ≥ 99%), acetaminophen ($C_8H_9NO_2$, ≥ 98%). Most of the chemicals used were of high purity and did not require further treatment, except for pyrrole, which required distillation. The synthesis reactions were carried out under nitrogen protection.

Instruments and apparatus

For the instrumentation used in this experiment, ^1H NMR spectra were taken on the Bruker DPX 400 spectrometer (400 MHz) in $CDCl_3$. FT-IR spectra were measured on a PerkinElmer Frontier™ infrared spectrometer ranging from 4000 to 500 cm^{-1} using pure potassium bromide for comparison. Raman spectra were recorded using a Thermo Fisher DXR Laser Microscopic Confocal Raman Spectrometer. The Shimadzu UV-2600 UV-vis spectrophotometer was used to measure the UV-vis diffuse reflectance spectrum of solid powders at room temperature. Powder X-ray diffraction (PXRD) patterns were obtained on a Rigaku Miniflex 600 X-ray powder diffractometer equipped with a Cu sealed tube (A= 1.540598 A) at a scan rate of 2

deg min'. The component of polymers obtained by X-ray photoelectron spectroscopy (XPS, Thermo Fisher Escalab Xi+, America). JSM-7610FPlus scanning electron microscope was used. The nitrogen adsorption and desorption isotherms were measured at 77 K using an BSD-PS surface area & pore size analyzer. The thermogravimetric analysis was performed on a Netzsch TG 209 F3 thermogravimetric analyzer with a ramp rate of 20 K min^{-1} and a temperature of 35 to 800 °C under the atmosphere of nitrogen. Electrochemical testing was performed using a China Shanghai Chenhua CHI660E workstation, a traditional three-electrode system consisting of a platinum mesh counter electrode, an Ag/AgCl reference electrode, and a glassy carbon electrode working electrode.

Preparation of 5,10,15,20-tetrakis(4-bromophenyl)-porphyrin (TBrPP)

TBrPP and CoTBrPP were synthesized according to the literature[1] and the synthesis process is shown in Scheme 1. p-Nitrobenzaldehyde (1.85 g, 10 mmol) was dissolved in propionic acid (100 mL) in a 250 mL flask and stirred at 140 °C for 10 min. Pyrrole (0.6928 mL, 10 mmol) was dissolved in 10 ml of propionic acid and added dropwise to the above solution through a constant-pressure dropping funnel over 10-15 min. After dropwise addition, the reaction mixture was stirred at 138 °C for 30 min. After the reaction was stopped, the reaction was cooled to room temperature, and a large amount of methanol was added and recrystallized in a refrigerator for 6-8 h. The crude product was washed with methanol after filtration under reduced pressure. Subsequently, the crude product was purified by column chromatography (the eluent was dichloromethane) to obtain 5,10,15,20-tetrakis(4-bromophenyl)-porphyrin (TBrPP). ^1H NMR ($CDCl_3$, 400 MHz): δ 8.87 (d, 8H, Ar-H), 8.07 (s, 8H, Ar-H), 7.93 (d, 8H, C=C-H), -2.84 (s, 2H, N-H).

Preparation of cobalt(II) 5,10,15,20-tetrakis(4-bromophenyl)-porphyrin (CoTBrPP)

TBrPP (0.50 g, 0.54 mmol) was dissolved in N,N-dimethylformamide (80 mL) in a 250 mL flask, and cobalt acetate tetrahydrate (1.0128 g, 4.55 mmol) was added and stirred for 8 h at 150 °C, protected by nitrogen throughout. The reaction was cooled to room temperature, the solvent was removed by distillation under reduced pressure,

and the remaining crude product was purified by column chromatography (the eluent was dichloromethane) and evaporated to obtain CoTBrPP as a purple solid.

Preparation of 5-Phenyldipyrromethane

Pyrrole (3.92 mL, 0.05 mol) and benzaldehyde (1.92 mL, 0.02 mmol) were added to 100 mL of distilled water containing 1.5 mL of hydrochloric acid and stirred under nitrogen for 90 min at room temperature under light, then filtered, washed five times each with water and petroleum ether in turn, purified by column chromatography (eluent dichloromethane: petroleum ether=1:1), and obtained by spin evaporation yielded white 5-Phenyldipyrromethane. ^{1}H NMR (400 MHz, $CDCl_3$): δ 7.82 (s, 2H), 7.32-7.24 (m, 4H), 6.62 (s, 2H), 6.13 (q, 2H), 5.89 (s, 2H), 5.42 (s, 1H).

Preparation of 5,15-di(4-[(trimethylsilyl)ethynyl]phenyl)-10,20-diphenylporphyrin (DETPP-TMS)

4-[(trimethylsilyl)ethynyl]benzaldehyde (421 mg, 1.48 mmol) and 5-Phenyldipyrromethane (350 mg, 1.48 mmol) were dissolved in CH_2Cl_2 (100 mL), and nitrogen bubbles were degassed for 30 min, followed by the addition of trifluoroacetic acid (77 μL, 1.04 mmol). After stirring at 25 °C for 3 h, 2,3-dichloro-5,6-dicyanobenzoquinone (680 mg, 2.96 mmol) was added. After stirring for 1.5 h, the reaction was quenched by adding triethylamine and detected by TLC. After vacuum distillation, purple DETPP-TMS solid was obtained by column chromatography purification on silica gel with CH_2Cl_2/petroleum ether (1:1, v/v). ^{1}H NMR (400 MHz, $CDCl_3$)：δ 8.89 (d, 8H, Ar-H), 8.22 (s, 8H, Ar-H), 7.91 (d, 4H, C=C-H), 7.78 (d, 6H, Ar-H), 0.40 (s, 18H, TMS), -2.79 (s, 2H, N-H).

Preparation of 5,15-di(4-ethynylphenyl)-10,20-diphenylporphyrin (H_2DETPP)

5,15-di(4-[(trimethylsilyl) ethynyl]phenyl)-10,20-diphenylporphyrin (100 mg, 0.13 mmol), K_2CO_3 (183 mg, 0.27 mmol) was dissolved in CH_2Cl_2/CH_3OH mixtures which were degassed by bubbling with nitrogen for 30 min and then stirred for 12 h at room temperature. The reaction was monitored by TLC analysis. After solvent removal, the resulting mixture was redissolved in CH_2Cl_2 (50 mL) and washed with water. Then, the resultant was purified by column chromatography on silica gel

eluting with petroleum ether/dichloromethane (1:1, v/v) to obtain 5,15-di(4-ethynylphenyl)-10,20-diphenyl porphyrin as a purple solid. ^{1}H NMR (400 MHz, $CDCl_3$): δ 8.87 (s, 8H, Ar-H), 8.22 (s, 8H, C=CH), 7.91 (s, 4H,C=CH), 7.76 (d, 6H, Ar-H), 3.35 (s, 2H, C≡CH), -2.77 (s, 2H, N-H).

Preparation of zinc(II) 5,15-di(4-ethynylphenyl)-10,20-diphenylporphyrin (ZnDETPP)

H_2DETPP (489.9 mg, 0.71 mmol), $Zn(OAc)_2{\cdot}2H_2O$ (1.1545 g, 5.26 mmol) was stirred in DMF at 150 °C for 6 h. After evaporation of the solvent, it was washed with deionised water, and purified by column chromatography on CH_2Cl_2 silica gel to obtain ZnDETPP.

Preparation of modified electrode

Glassy carbon electrodes (GCE) with a 3 mm diameter were polished sequentially: first with a nylon polishing cloth, then to a mirror finish using 0.05 μm aluminum oxide. After thorough rinsing with deionized water, the electrodes were air-dried and set aside. The 5 mg CoTBrPP-ZnDETPP sample was dispersed in 800 μL of anhydrous ethanol and subjected to ultrasonic treatment for 30 minutes to form a suspension. To prepare the CoTBrPP-ZnDETPP-modified electrode, 10 μL of this suspension was pipetted onto the cleaned electrode surface and allowed to air-dry at room temperature. The CoTBrPP-H_2DETPP and TBrPP-ZnDETPP-modified electrodes were prepared similarly.

Electrochemical measurements

In this work, APAP detection was analyzed in 0.1 M phosphate buffer solution (PBS) utilizing CV, DPV, and electrochemical impedance spectroscopy (EIS). The CV and DPV experiments were carried out at a 100 mV s^{-1} scan rate across a potential window of -0.2 V to 0.6 V. EIS analysis was performed over a frequency range spanning from 0.01 Hz to 10^6 Hz.

Figures

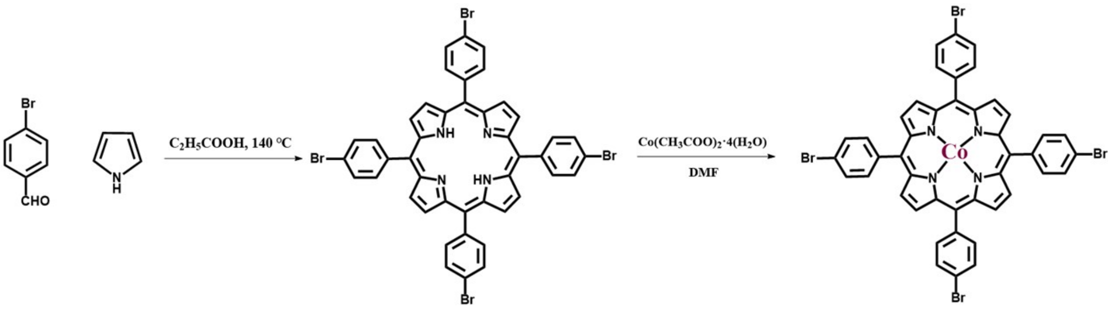

TBrPP CoTBrPP

Fig. S1 Preparation of TBrPP and CoTBrPP.

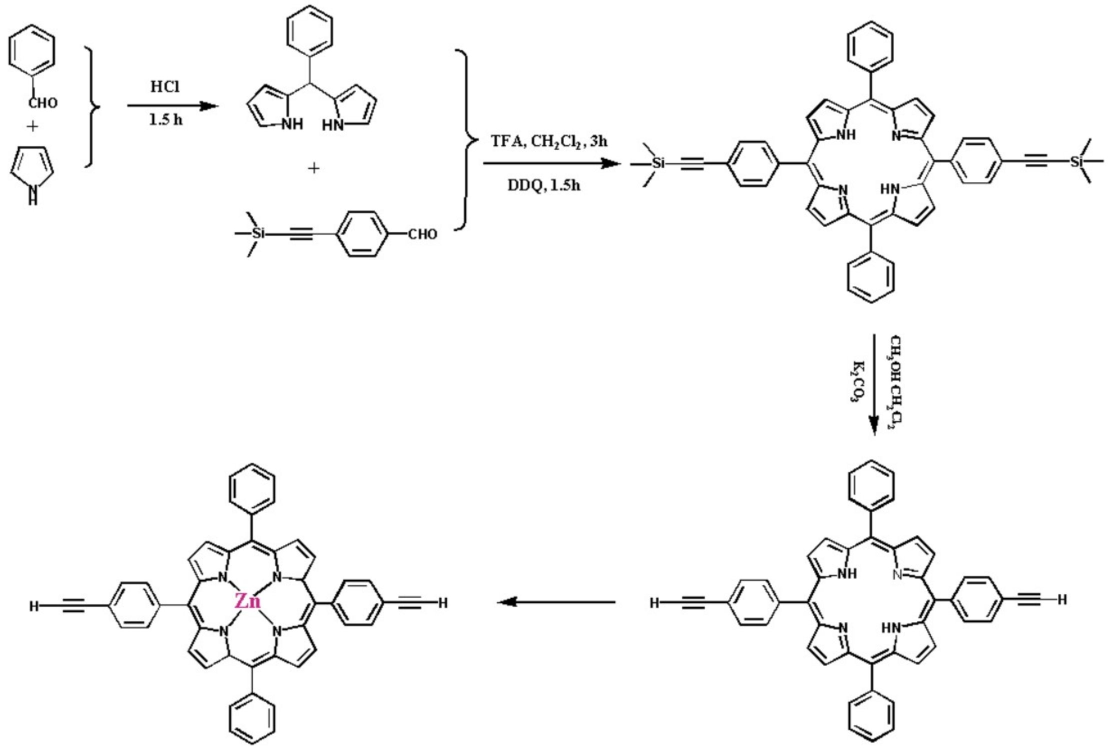

Zn-DETPP

Fig. S2 Preparation of ZnDETPP.

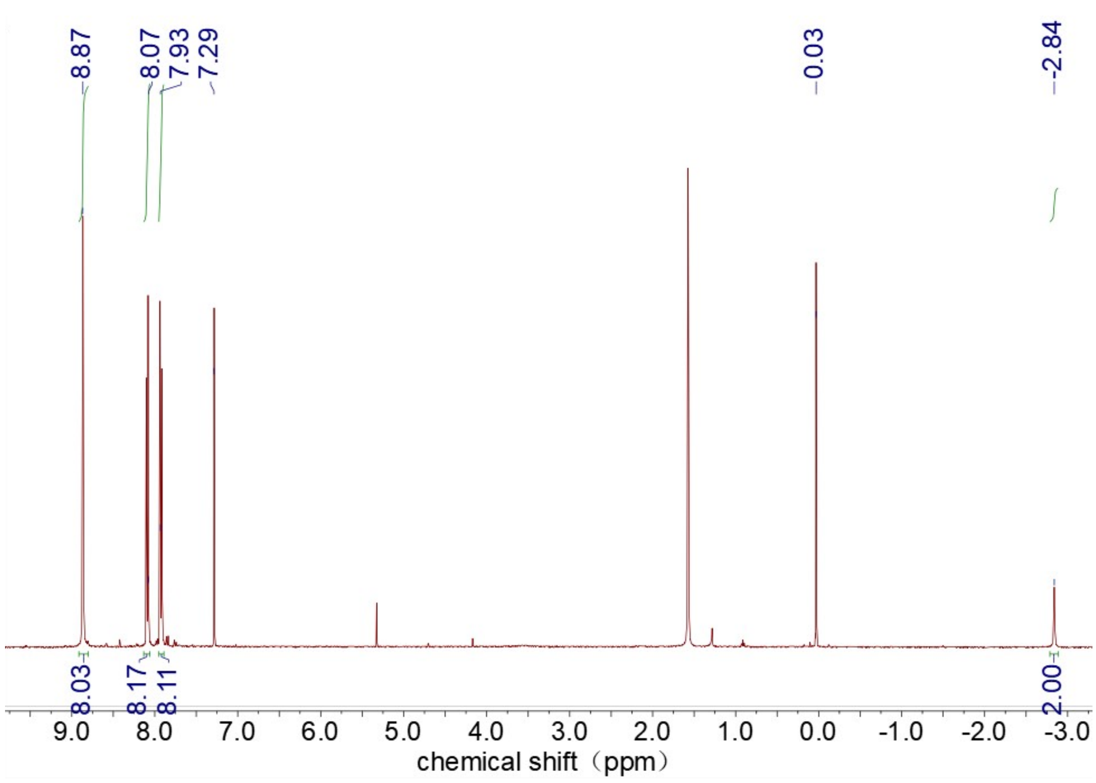

Fig. S3 ^{1}H NMR (in $CDCl_3$) spectrum of TBrPP.

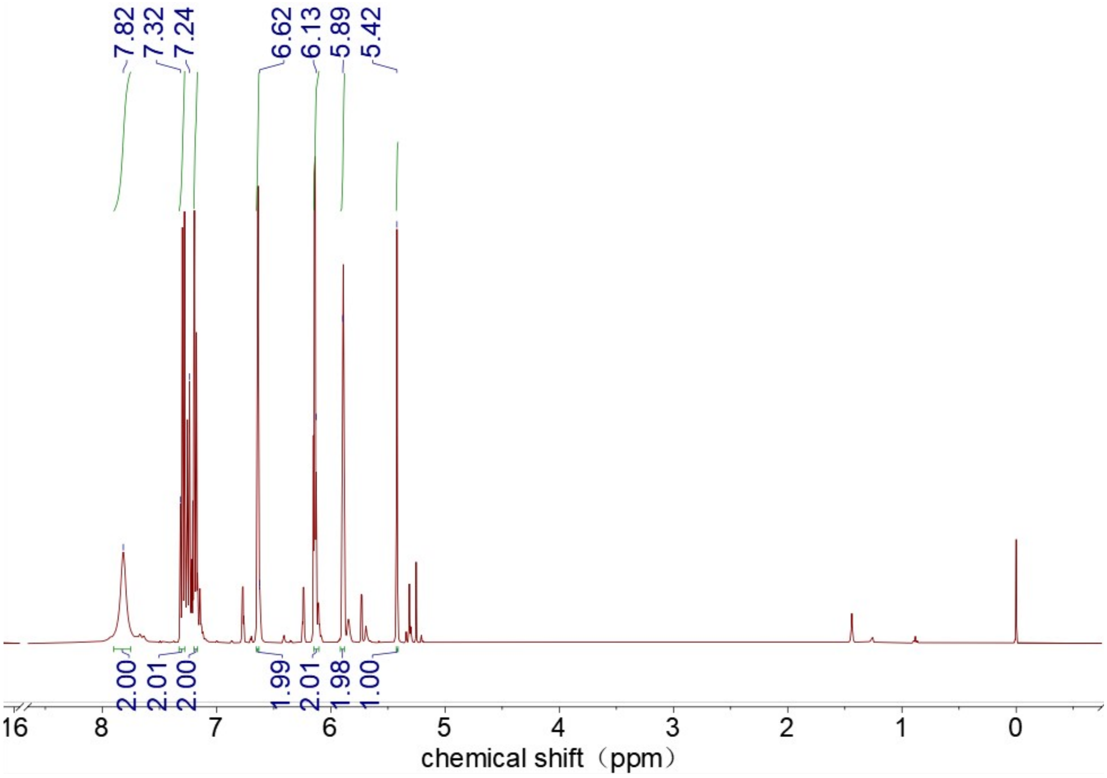

Fig. S4 ^{1}H NMR (in $CDCl_3$) spectrum of 5-Phenyldipyrromethane.

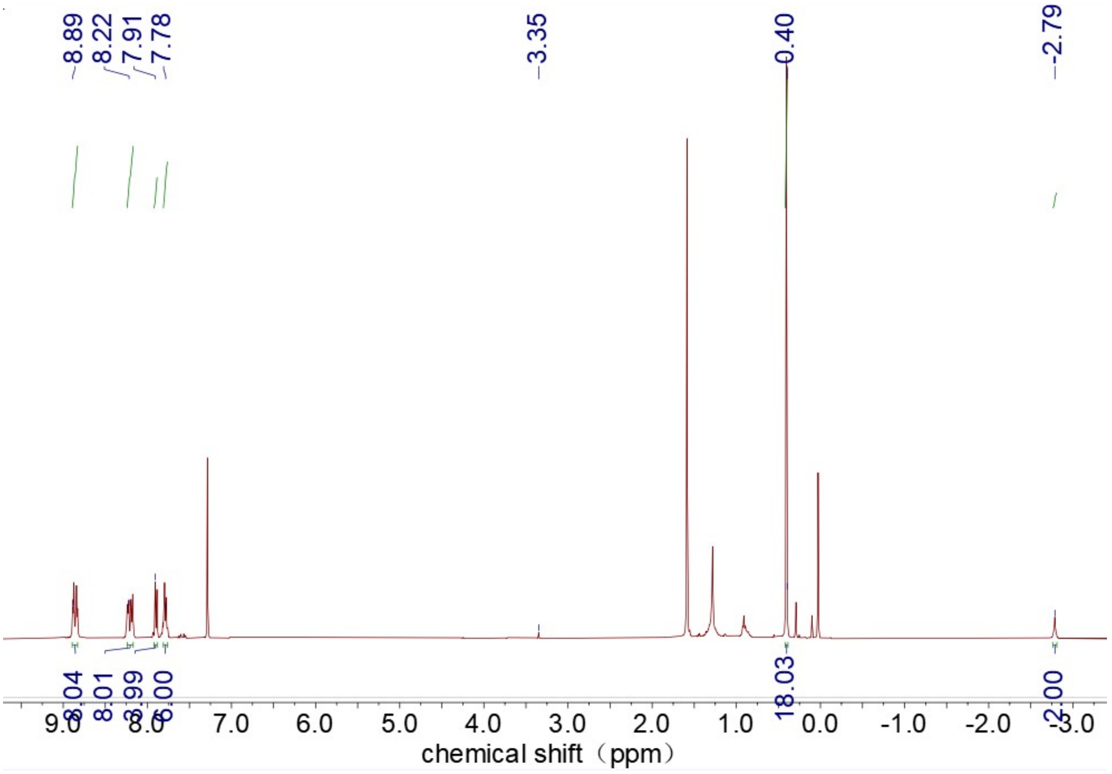

Fig. S5 ^{1}H NMR spectrum of DETPP-TMS.

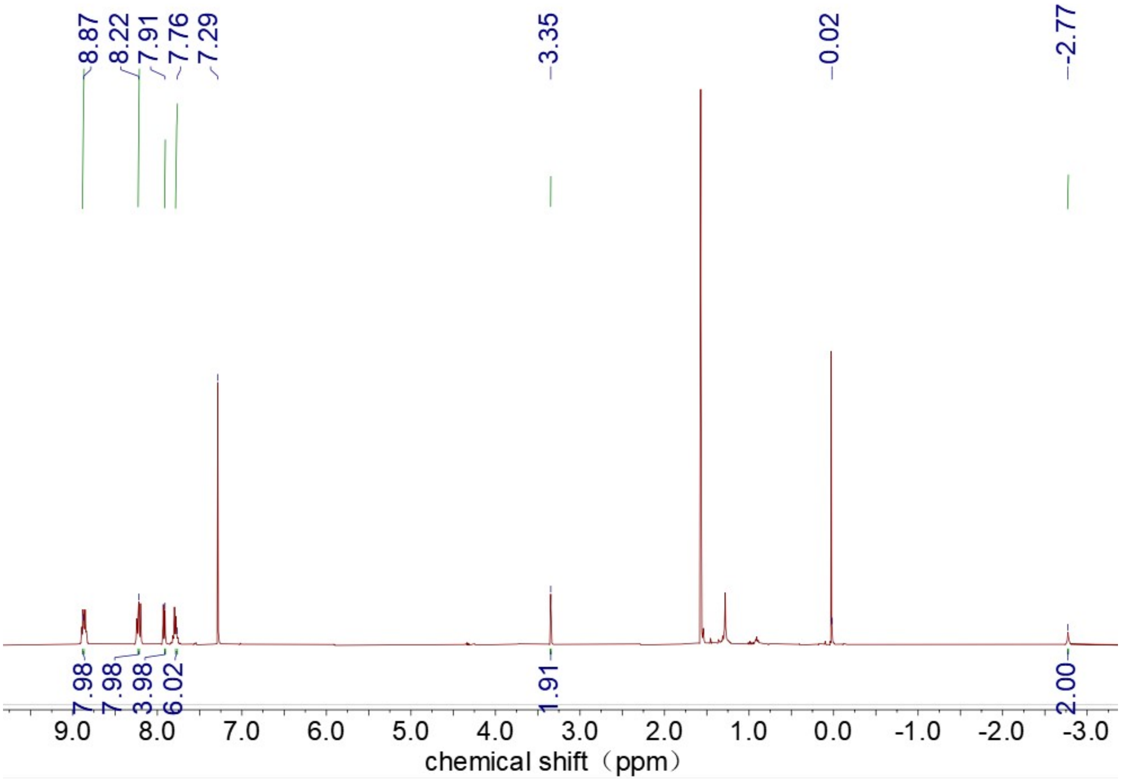

Fig. S6 ^{1}H NMR spectrum of H_2DETPP.

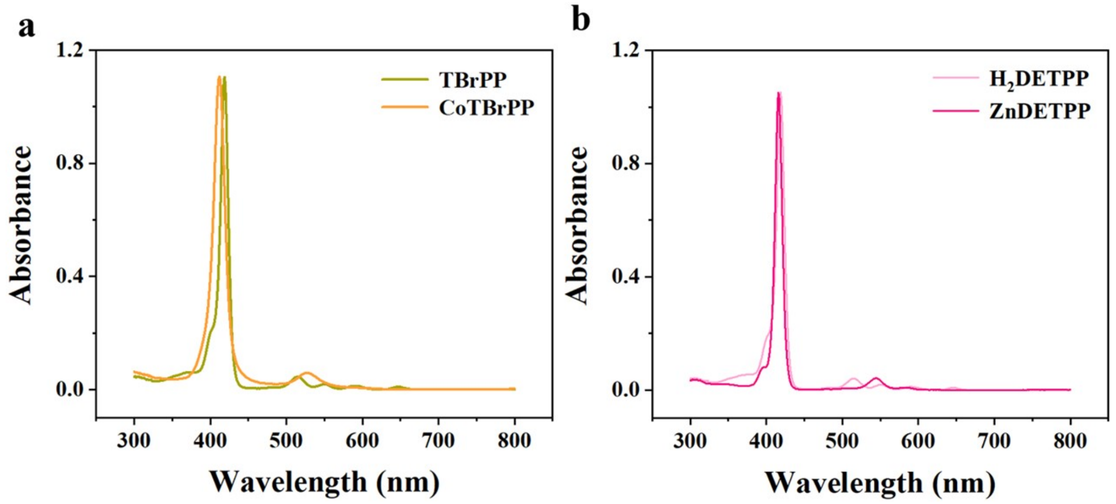

Fig. S7 UV-vis absorption spectra of TBrPP and CoTBrPP (a), H_2DETPP and ZnDETPP (b).

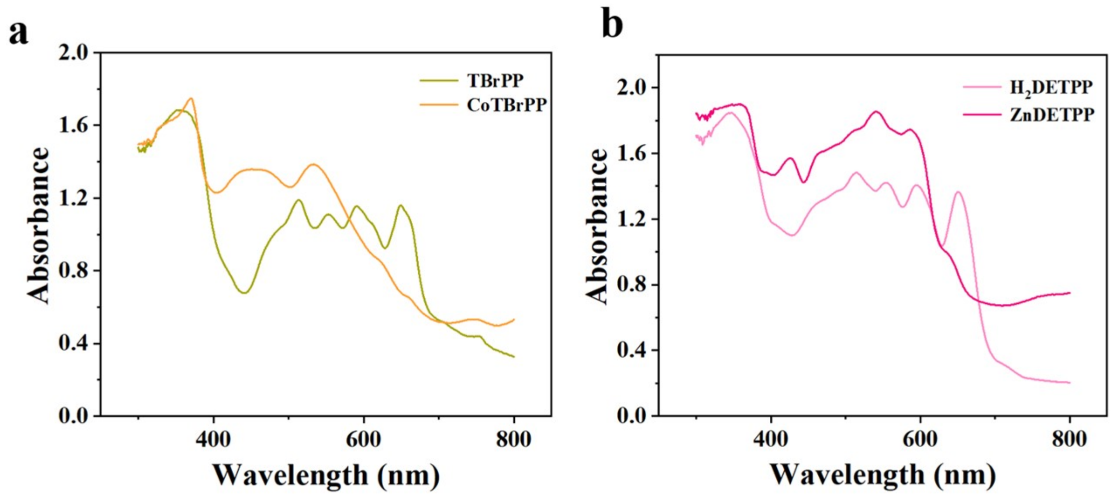

Fig. S8 UV–vis diffuse reflectance spectra of TBrPP and CoTBrPP (a), H_2DETPP and ZnDETPP (b).

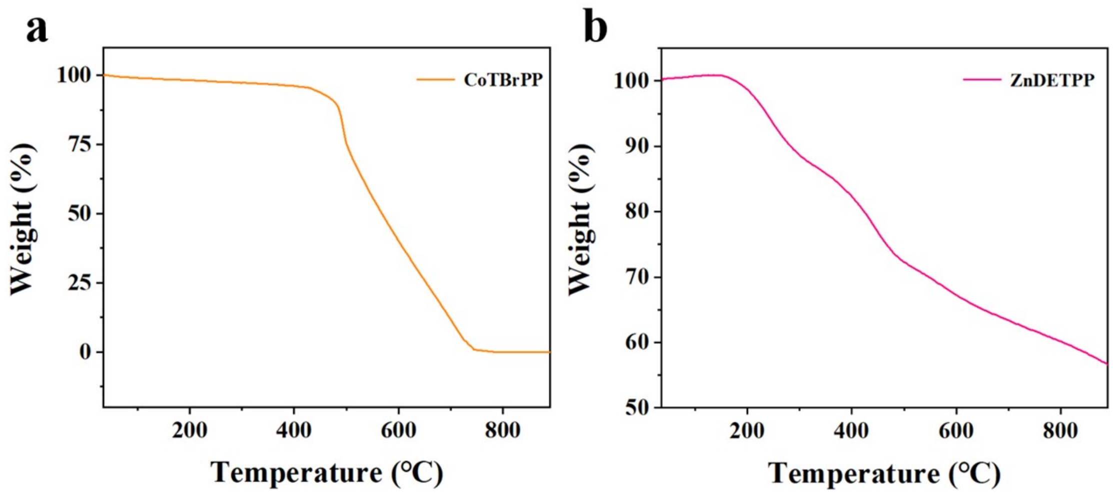

Fig. S9 TGA of CoTBrPP (a), ZnDETPP (b).

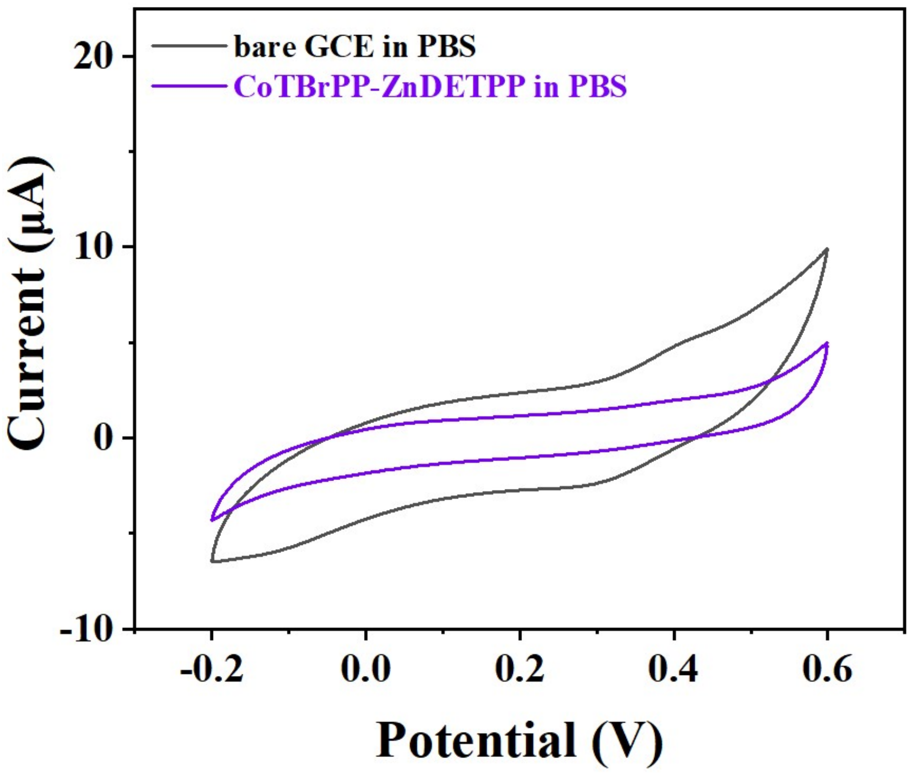

Fig. S10 CV detection curves of bare GCE and CoTBrPP-ZnDETPP/GCE electrode in 0.1 M PBS buffer solution.

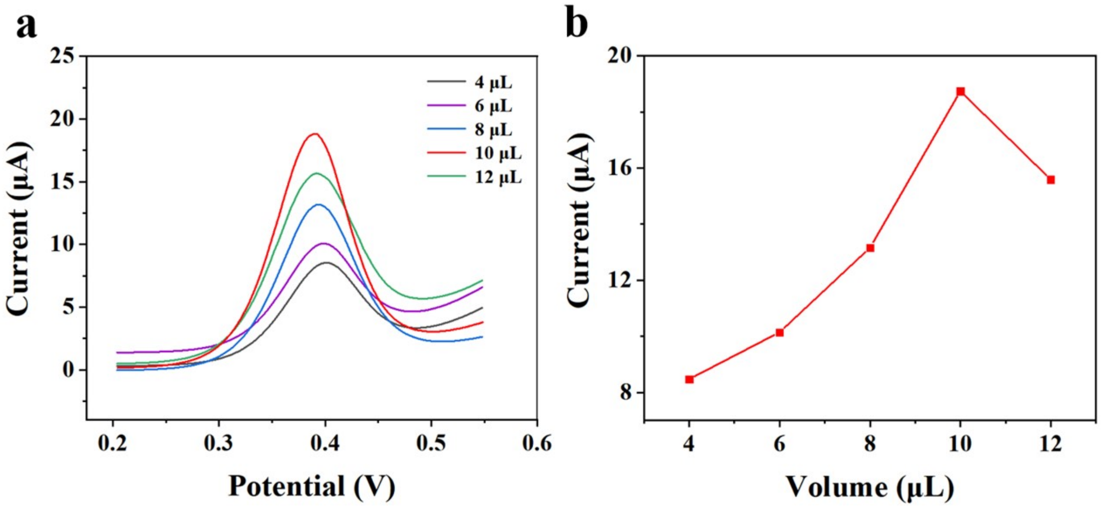

Fig. S11 Spotting volume on the peak current response for 1 mM APAP in 0.1 M PBS (a) and the effect of volume value on the current (b).

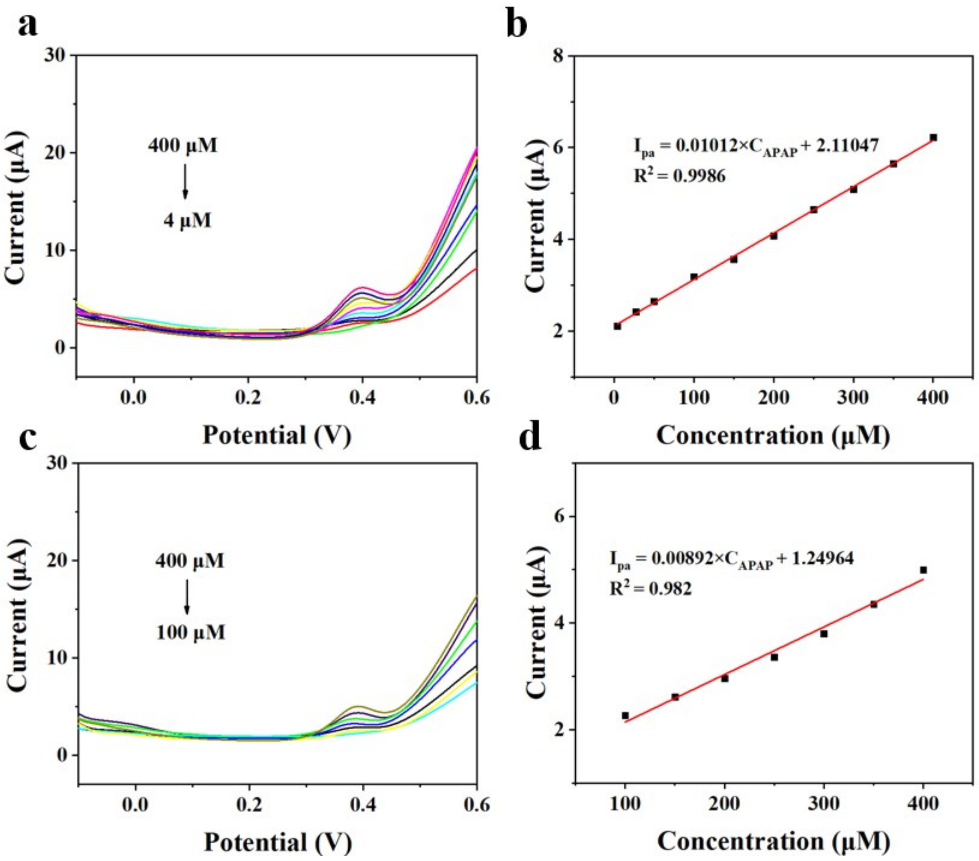

Fig. S12 The DPV signals at CoTBrPP-ZnDETPP/GCE of APAP with different concentrations (4~400 µM) in PBS (a), The peak current intensity vs. The concentrations of APAP (b), The DPV signals at TBrPP-ZnDETPP/GCE of APAP with different concentrations (100~400 µM) in PBS (c), and the peak current intensity vs. The concentrations of APAP (d).

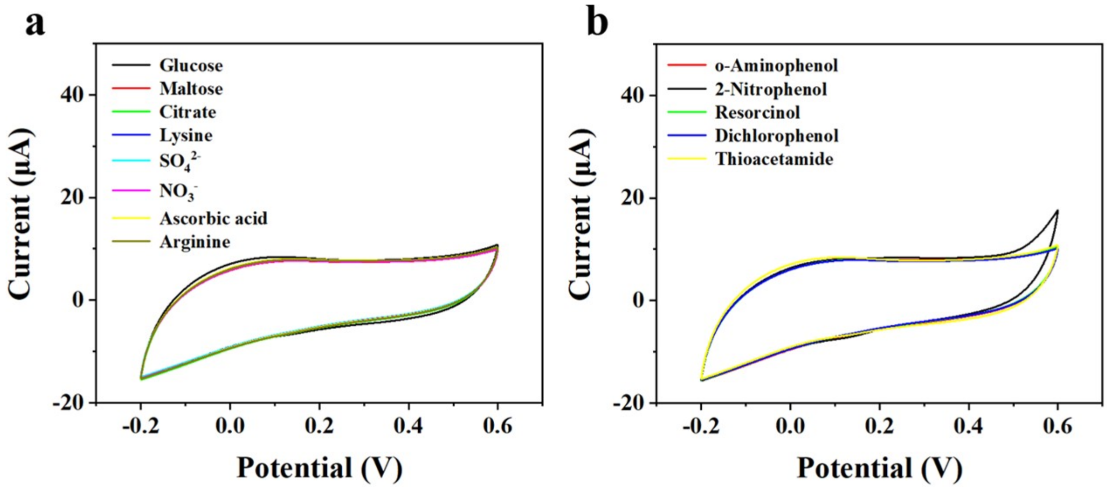

Fig. S13 CVs of competitive molecules (a) and interfering substances similar to the structure of APAP (b) detected by GCE.

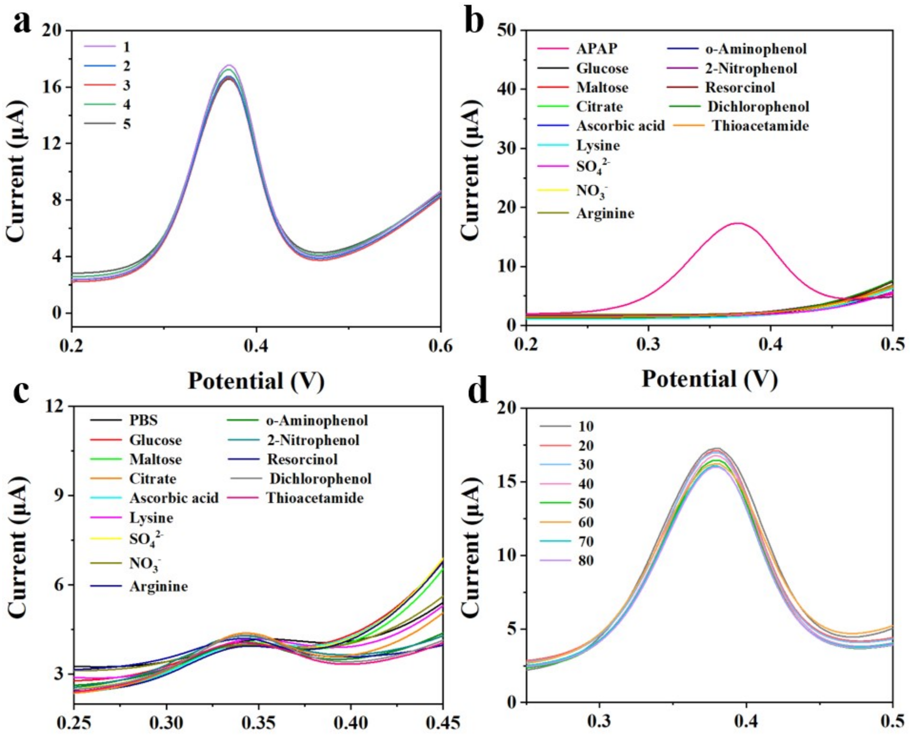

Fig. S14 The reproducibility (a), selectivity (b), interference (c) and stability (d) of CoTBrPP-ZnDETPP/GCE sensor.

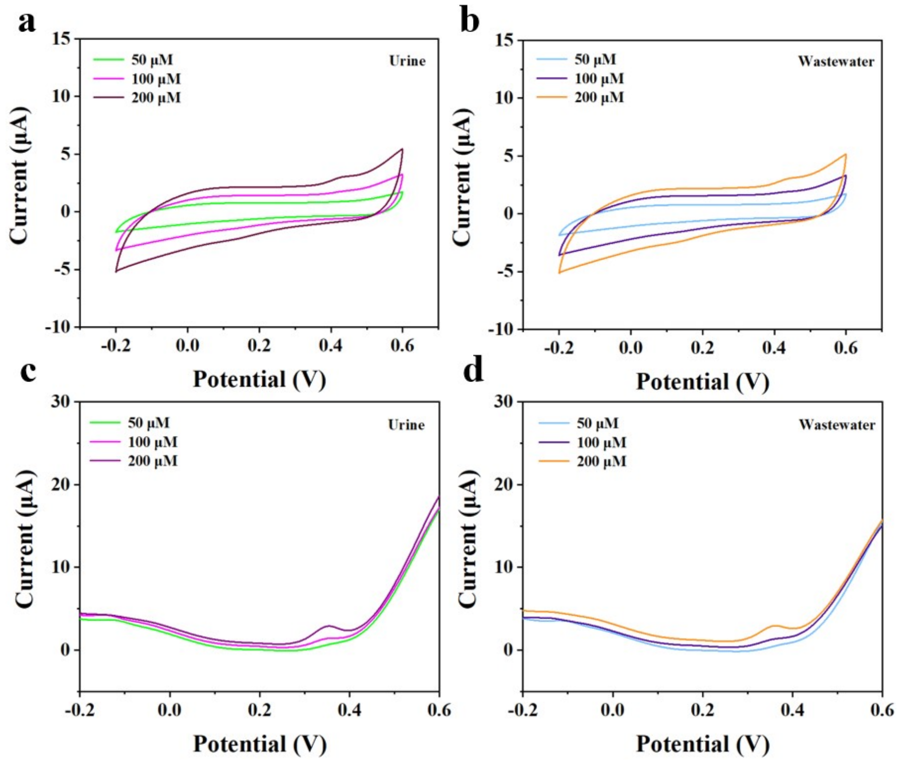

Fig. S15 CV of APAP determination by modified electrode in urine (a) and wastewater (b), DPV of APAP determination by modified electrode in urine (c) and wastewater(d).

References

1 Z. Q. Chen, J. M. Wang, S. Zhang, Y. X. Zhang, J. Zhang, R. J. Li and T. Y. Peng, *ACS Appl. Energy Mater.*, 2019, **2**, 5665-5676.
